# Causal association between thyroid dysfunction and sepsis: a two-sample mendelian randomization study

**DOI:** 10.3389/fendo.2024.1348248

**Published:** 2024-03-22

**Authors:** Junbin Hong, Lian Zhang, Yanni Lai, Xinying Chen, Yiting Chen, Jinghua Yang

**Affiliations:** ^1^ The Second Clinical Medical College, Guangzhou University of Chinese Medicine, Guangzhou, China; ^2^ School of Medicine and Health, Shunde Polytechnic, Foshan, China; ^3^ Department of Pediatrics, The Second Affiliated Hospital, Guangzhou University of Chinese Medicine, Guangzhou, China; ^4^ Xiaorong Luo’s National Renowned Expert Inheritance Studio, Guangdong Provincial Hospital of Chinese Medicine, Guangzhou, China

**Keywords:** hyperthyroidism, hypothyroidism, mendelian randomization, sepsis, thyroid dysfunction

## Abstract

**Background:**

The causal association between thyroid dysfunction (including hyperthyroidism and hypothyroidism) and sepsis is controversial in previous studies. Therefore, we used Mendelian randomization (MR) to explore the causal association between hyperthyroidism or hypothyroidism and the susceptibility to four distinct subtypes of sepsis (streptococcal sepsis, puerperal sepsis, asthma-associated pneumonia or sepsis, and other sepsis).

**Methods:**

In our research, we conducted two-sample Mendelian randomization (MR) analyses utilizing publicly available genome-wide association studies (GWAS) data from Sakaue et al. and the Finnish database to investigate the potential causal associations between hyperthyroidism, hypothyroidism, and each of the four distinct subtypes of sepsis, in addition to reverse MR analyses of the positive results to examine the existence of reverse causality.

**Results:**

Genetic hypothyroidism was causally related to the development of asthma-associated pneumonia or sepsis (OR_IVW_: 1.097, 95% CI: 1.024 to 1.174, *P* = 0.008); hypothyroidism was significantly associated with the development of other sepsis (OR_IVW_: 1.070, 95% CI: 1.028 to 1.115, *P* < 0.001). In addition, sensitivity analysis substantiated the robustness of these two MR findings, with no evidence of horizontal pleiotropy observed (*P* > 0.05). MR Egger regression analysis demonstrated no heterogeneity between instrumental variables (IVs). Inverse MR results confirmed no reverse causality between hypothyroidism and asthma-associated pneumonia or sepsis, or between hypothyroidism and other sepsis. The findings of this study also unveiled that there is no evidence of a causal link between hypothyroidism and the development of streptococcal sepsis or puerperal sepsis. Additionally, the research provided evidence indicating the absence of a causal relationship between hyperthyroidism and streptococcal sepsis, puerperal sepsis, asthma-associated pneumonia or sepsis, and other sepsis.

**Conclusions:**

This study identified a causal link between hypothyroidism and the occurrence of asthma-associated pneumonia or sepsis, and other sepsis, but not with the development of streptococcal sepsis and puerperal sepsis. Moreover, our findings did not reveal any causal association between hyperthyroidism and streptococcal sepsis, puerperal sepsis, asthma-associated pneumonia or sepsis, and other sepsis.

## Introduction

Sepsis, a potentially fatal syndrome characterized by organ dysfunction caused by an imbalanced response to infection, is a prominent global health concern. It imposes a substantial burden on healthcare systems worldwide, with rising incidence and mortality rates observed on a global scale (1, 2). The pathogenesis of sepsis involves complex dysregulation of the immune system, abnormal inflammatory responses, cellular signaling transduction at multiple levels. The intricate nature of this disease presents significant challenges in terms of its prevention and treatment (3).

Thyroid dysfunction refers to the abnormal synthesis and release of thyroid hormones (TH), including thyroxine (T4), triiodothyronine (T3), etc., triggering a disruption in the metabolic regulation of body. It encompasses two types: hyperthyroidism and hypothyroidism (4), which have a significant global prevalence. However, approximately half of thyroid dysfunction cases remain undiagnosed (5). The physiological and pathological processes between the endocrine system and the immune system in patients with thyroid dysfunction mutually interact and influence each other. Dysfunctions in either system can contribute to the development of diseases including sepsis, inflammation, autoimmune disorders, among others (6).

The causal association between thyroid dysfunction and sepsis has been a subject of controversy previously. Some studies suggested that patients with sepsis may develop secondary thyroid dysfunction as a result of the compensatory response of body (7, 8). A study has shown that hypothyroidism can abolish the hyperdynamic phase of sepsis, leading to increased susceptibility and mortality rates (9). Additionally, the other research has indicated a potential role for TH in modulating muscle protein metabolism during sepsis (10). However, there is also another study that indicate no significant differences in terms of mortality or 30-day readmission outcomes between sepsis patients with hypothyroidism and those with normal thyroid function (11). These studies suggest the controversial nature of the causal association between thyroid dysfunction and sepsis, thus emphasizing the need for further research.

Mendelian randomization (MR) is a method that integrates summary data from genome-wide association studies (GWAS) to minimize the influence of reverse causation and confounding factors. It is widely used to assess the existence of causal association and evaluate associations between exposures and complex outcomes. MR has emerged as a crucial research method for enhancing our understanding, prevention, and treatment of thyroid disease and sepsis (12, 13). Therefore, we explored the causal association between thyroid dysfunction and streptococcal sepsis, puerperal sepsis, asthma-associated pneumonia or sepsis, and other sepsis, respectively, by taking advantage of a two-sample MR approach with genetic IVs, to provide compelling evidence for the prevention of sepsis among patients with thyroid dysfunction.

## Methods

### Data sources of exposure and outcome

The GAWS in our research is derived from Sakaue et al.’s research findings (14) and the Finnish database (15). To identify IVs, we conducted a thorough screening of single nucleotide polymorphisms (SNPs) significantly correlated with thyroid dysfunction. By leveraging the screened SNPs, several MR methods were exploited to investigate the causal link between thyroid dysfunction and sepsis. **Table 1** provides a comprehensive overview of the summary information derived from the GWAS data applied in our research.

**Table 1:** Summary information on the GWAS data in this study.

Traits	Data source	Population	Case	Control	Sample size	Years
Hyperthyroidism	A cross-population atlas of genetic associations for 220 human phenotypes	Mixed	3,557	456,942	460,499	2021
Hypothyroidism	A cross-population atlas of genetic associations for 220 human phenotypes	Mixed	30,155	379,986	410,141	2021
Streptococcal septicaemia	FinnGen	European	2,348	332,343	334,691	2021
Puerperal sepsis	FinnGen	European	3,940	202,267	206,207	2021
Asthma-related pneumonia or septicaemia	FinnGen	European	5,545	135,449	140,994	2021
Other sepsis	FinnGen	European	12,301	332,343	3446,44	2021

To ensure the credibility and precision of the causal association conclusion between thyroid dysfunction and the risk of developing sepsis, we implemented meticulous quality control procedures during the selection of genetic IVs. First, we identified genetic SNPs variants that exhibit genome-wide significance in relation to hyperthyroidism and hypothyroidism. Data is filtered based on *P*-values and linkage disequilibrium (LD) pruning (*P<* 5×10^-8^, LD: *r^2^<* 0.001, KB = 10,000). Subsequently, we calculated the *F*-values of the selected SNPs using the formula $F=\left(\text{N}-2\right)\times \frac{R^{2}}{1-R^{2}}$ , where *N* represents the sample size and *R^2^* refers to the squared correlation coefficient. IVs with *F*-values>10 are considered strongly correlated with the exposure. The formula to calculate *R^2^* in this context is: $R^{2}=2\times (1-\text{EAF})\times \text{EAF}\times (\frac{\beta }{SE\times \sqrt{\text{N}}}{)}^{2}$ . I n the provided formula, *N* represents the sample size of the selected dataset, *β* refers to the effect size of the SNPs on the exposure, *SE* is the standard error of *β*, and EAF stands for the effect allele frequency. Furthermore, we utilized the phenoscanner database to search for relevant phenotypes associated with the remaining SNPs to exclude SNPs that showed significant associations with phenotypes related to sepsis outcomes. Additionally, we extracted data on the sepsis outcome phenotypes from GWAS studies and examined the association between the SNPs meeting the assumptions described below and the sepsis outcomes. After that, we merged the exposure and outcome datasets, which included both IVs and their association with the outcomes and exposures. Any duplicate or redundant SNPs were eliminated. The remaining SNPs comprised the definitive set of IVs that indicated the exposure.

### Description of study design

**Figure 1:**
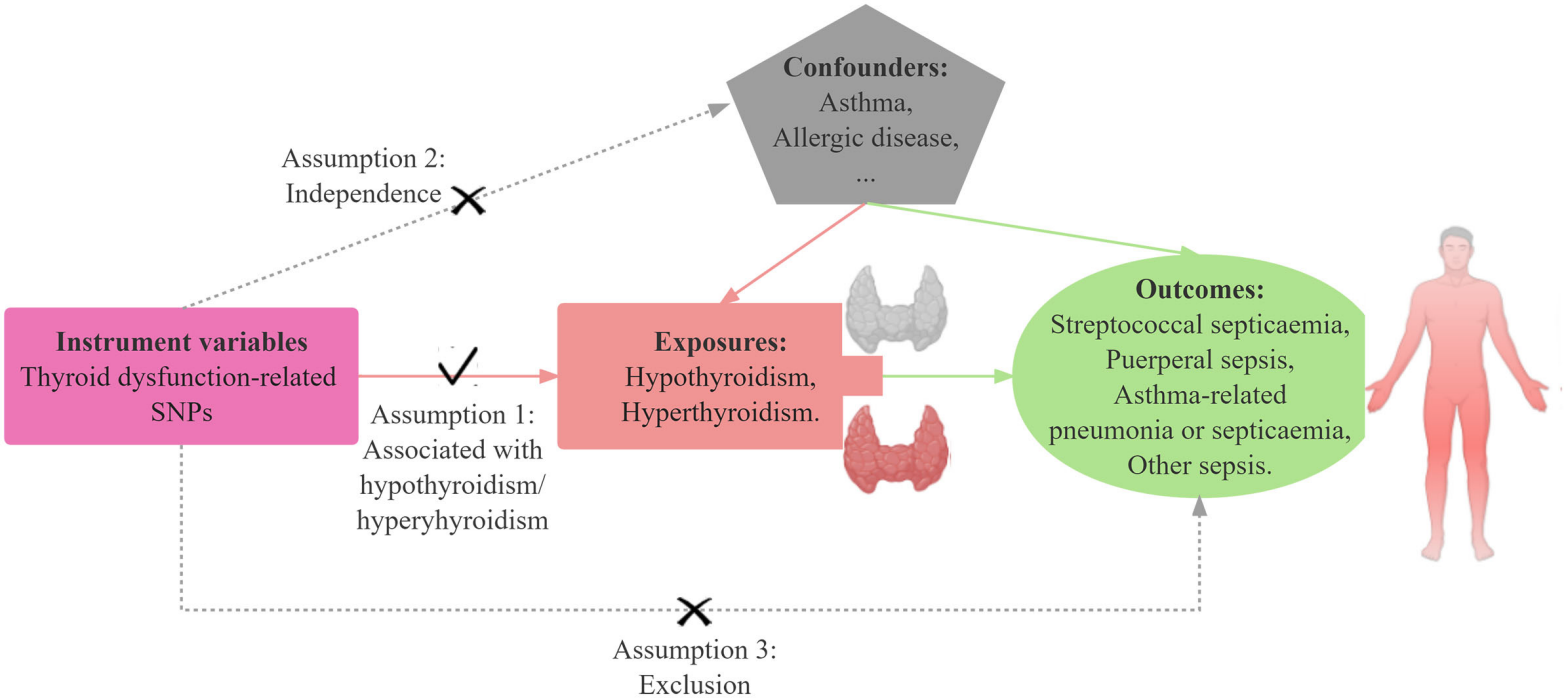
Schematic model of the Mendelian randomization (MR) study. SNPs, single-nucleotide polymorphisms. Solid arrows represent valid relationships between variables based on the assumptions of MR, while dashed lines indicate relationships that are not allowed for valid instrumental variables (IVs).

**Figure 2:**
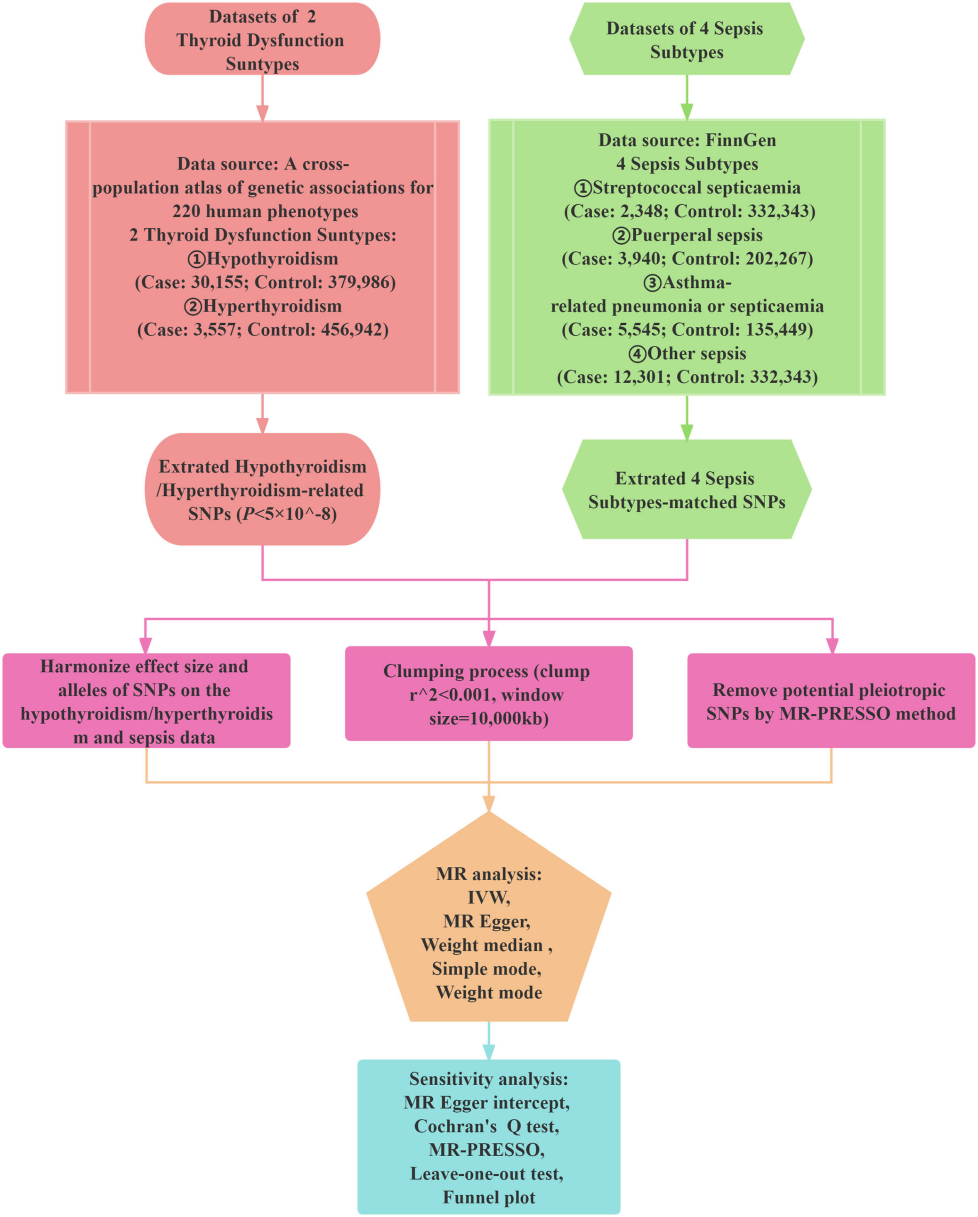
Study design of the Mendelian randomization (MR) study on the associations of hypothyroidism/hyperthyroidism and sepsis. SNPs, single nucleotide polymorphisms; MR-PRESSO, MR pleiotropy residual sum and outlier; IVW, Inverse Variance Weighted.

To explore the possible causal association between thyroid dysfunction and sepsis, a two-sample MR study was conducted using Inverse Variance Weighting (IVW), MR-Egger, Weighted Median, Simple Mode, Weighted Mode five statistical approaches with hyperthyroidism and hypothyroidism as exposures, the four sepsis subtypes as outcomes. The SNPs used as IVs in this research must satisfy three assumptions: a) Relevance assumption: the SNPs should exhibit a strong correlation with the exposure variable.; b) Exclusion restriction assumption: the SNPs should have a sole impact on the outcome variable through their association with the exposure variable, without any influence through alternative pathways.; c) Independence assumption: the SNPs are unrelated to the outcome variable and are not influenced by any other confounding factors that may affect the outcome. Subsequently, we additionally performed a sensitivity analysis to assess the robustness of the results obtained from the MR analyses. Heterogeneity test was first performed utilizing Cochran’s Q test, and then the test of multiplicity was realized utilizing leave-one-out analysis, funnel plot, MR-Egger intercept analysis, and MR PRESSO global test. **Figure 1** illustrates a schematic diagram of the MR causality study design, elucidating the fundamental principles of MR studies. **Figure 2** is a flow chart, illustrating the steps involved in the study.

### Statistical analyses

#### MR analyses

We conducted two sample MR analyses employing five statistical methods aforementioned to explore the possible causal association between thyroid dysfunction and sepsis. As the IVW is currently considered to be the most robust method of MR analyses, it was utilized as the primary basis for determining the outcome, while the other four methods as supplementary analyses. The five methods make distinct assumptions regarding horizontal pleiotropy. The IVW has the characteristic of not considering the presence of intercept term during regression and using the inverse of outcome variance (*SE* squared) as weights for fitting. This approach helps to mitigate bias caused by endogeneity. In the IVW, we used random effects as its main model, and the overall estimates derived from this method are comparable to a weighted linear regression of the Wald estimates for each SNP (16). The other four methods were employed because they offer more robust estimates in a broader range of situations. However, it is crucial to acknowledge that these methods have the potential to yield a smaller effect size, indicated by wider confidence intervals (17). After excluding SNPs that were directly associated with the outcome or exhibited horizontal pleiotropy, MR-Egger was employed to conduct a valid test of causal effects consistent with the IVW method (18). Weighted Median is characterized by giving plausible estimates in situations where up to 50% of the results originate from invalid SNPs (19). If the majority of individual SNP causal effect estimates are derived from valid SNPs, the weighting pattern remains consistent even when some of the SNPs are invalid (20). In addition, Simple Mode is an unweighted approach used for causal estimation that relies on the empirical density function (21). Finally, to demonstrate the absence of reverse causality, we also performed reverse MR analyses of hypothyroidism and asthma-associated pneumonia or sepsis, and other sepsis.

#### Sensitivity analyses

We used the intercept term of the MR-Egger to evaluate the presence of horizontal pleiotropy, where *P* > 0.05 indicates a weak likelihood of heterogeneity in the causal analysis (17). Moreover, we applied the mendelian randomized pleiotropic residuals and outliers (MR-PRESSO) to determine horizontal pleiotropy and correct potential outliers, and MR analyses was performed again after screening outliers. For the heterogeneity test, we employed Cochran’s Q-test (22) and funnel plots to estimate the heterogeneity of IVW. Additionally, to evaluate the impact of individual SNPs on the primary causal associations, we conducted a leave-one-out analysis by systematically removing each SNP one at a time.

## Results

### Selection of IVs

In the MR analyses of hypothyroidism and asthma-associated pneumonia or sepsis, 59 IVs associated with hypothyroidism were ultimately included, and in the MR analysis of hypothyroidism and other sepsis, 57 IVs related to hypothyroidism were eventually incorporated, with *F*-values greater than 10 (**Supplementary Table S1**).

### MR analyses

**Figure 3:**
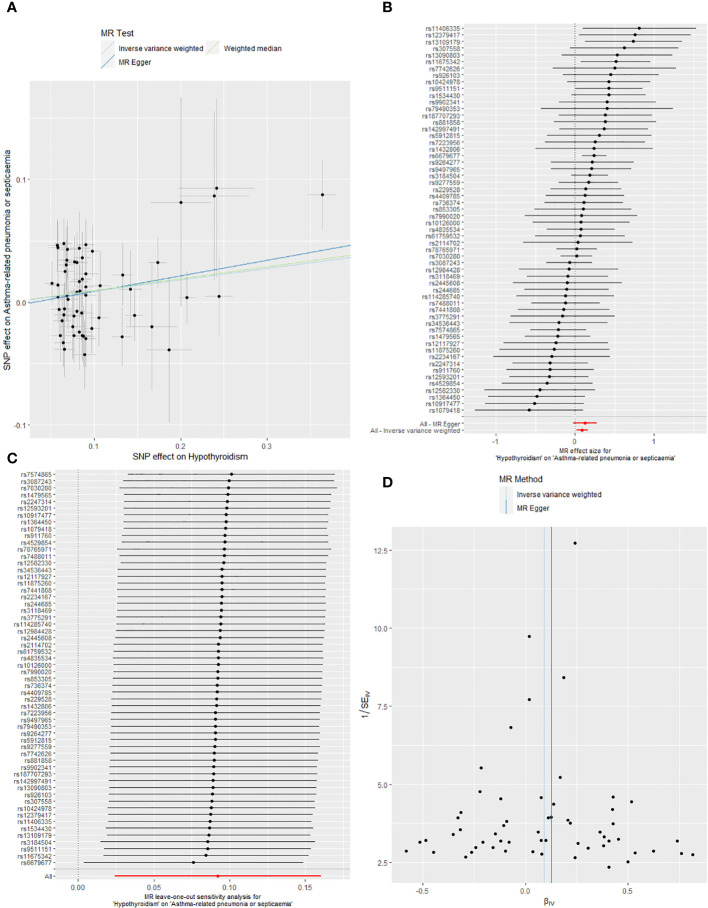
**(A)** Scatter plots of the causality between hypothyroidism and asthma-related pneumonia or sepsis by five MR analyses. **(B)** Forest plot of two-sample MR estimates the effects of hypothyroidism on asthma-related pneumonia or sepsis. **(C)** The leave-one-out sensitivity plot between hypothyroidism and asthma-related pneumonia or sepsis. **(D)** Funnel plots of significant and nominal significant estimates from genetically predicted hypothyroidism on asthma-related pneumonia or sepsis.

IVW analysis demonstrated that hypothyroidism was causally associated with the development of asthma-associated pneumonia or sepsis (OR: 1.097, 95% CI: 1.024 to 1.174, *P* = 0.008), and the four methods were negative, but the OR > 1, which was in the same direction as the results of the IVW (**Table 2**). **Figure 3A** showcases the causality between hypothyroidism and asthma-related pneumonia or sepsis by five MR analyses.

**Table 2:** MR analysis results for the association of hypothyroidism with asthma-related pneumonia or septicaemia.

Exposure/outcome	Methods	nSNP	OR (95%CI)	*P*-value	Test of heterogeneity		Intercept term			Global test	
					Q	*P*-value	Intercept	SE	*P*-value	RSSobs	*P*-value
Hypothyroidism/Asthma- related pneumonia or septicaemia	MR Egger	58	1.135(0.997,1.318)	0.103	74.790	0.065	-0.004	0.008	0.614	78.463	0.061
	WMe	58	1.101(0.993,1.122)	0.067							
	IVW	58	1.097(1.024,1.174)	0.008	72.133	0.060					
	SM	58	1.070(0.854,1.340)	0.559							
	WMo	58	1.141(0.996,1.308)	0.063							

nSNP, numbers of single-nucleotide polymorphism; WMe, Weighted Median; IVW, Inverse Variance Weighted; SM, Simple Mode; WMo, Weighted Mode.

**Figure 4:**
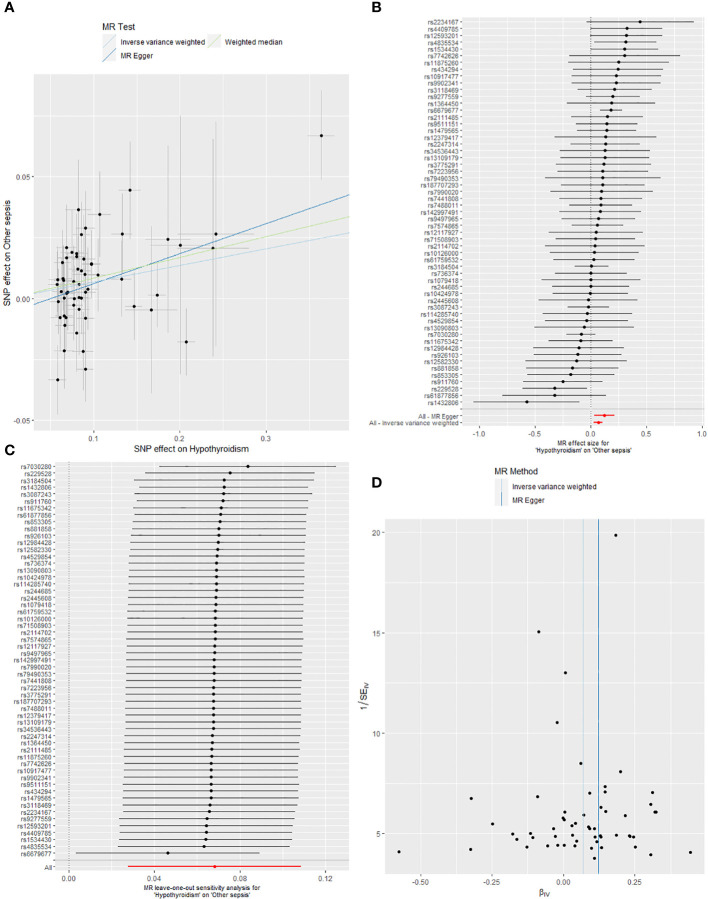
**(A)** Scatter plots of the causality between hypothyroidism and other sepsis by five MR analyses. **(B)** Forest plot of two-sample MR estimates the effects of hypothyroidism on other sepsis. **(C)** The leave-one-out sensitivity plot between hypothyroidism and other sepsis. **(D)** Funnel plots of significant and nominal significant estimates from genetically predicted hypothyroidism on other sepsis.

IVW analysis additionally showed that hypothyroidism was significantly connected to the occurrence of other sepsis (OR_IVW_: 1.070, 95% CI: 1.028 to 1.115, *P<* 0.001); furthermore, Weighted Median and MR Egger also manifested that hypothyroidism was causally related to the susceptibility of other sepsis, with Weighted Median showed OR: 1.089, 95% CI: 1.022 to 1.160, *P* = 0.008, and the MR Egger manifested OR: 1.131, 95% CI: 1.032 to 1.239, *P* = 0.011. Although the results of Simple Mode and Weighted Mode were negative, the OR > 1, which was in the same direction as the results of other methods (**Table 3**). The causal link between hypothyroidism and other sepsis through five MR analyses is emerged in **Figure 4A**.

**Table 3:** MR analysis results for the association of hypothyroidism with other sepsis.

Exposure/outcome	Methods	nSNP	OR (95%CI)	*P*-value	Test of heterogeneity		Intercept term			Global test	
					Q	*P*-value	Intercept	SE	*P*-value	RSSobs	*P*-value
Hypothyroidism/Other sepsis	MR Egger	57	1.131(1.032,1.239)	0.011	57.561	0.381	-0.006	0.005	0.195	64.090	0.295
	WMe	57	1.089(1.022,1.160)	0.009							
	IVW	57	1.070(1.028,1.115)	<0.001	59.362	0.354					
	SM	57	1.092(0.944,1.264)	0.241							
	WMo	57	1.168(1.008,1.353)	0.044							

nSNP, numbers of single-nucleotide polymorphism; WMe, Weighted Median; IVW, Inverse Variance Weighted; SM, Simple Mode; WMo, Weighted Mode.

The forest plots (**Figures 3B**, **4B**) display the causal effect estimates of hypothyroidism on asthma-associated pneumonia or sepsis, and other sepsis generated by the IVW and MR-Egger methods in MR analyses.

This study also unveiled that there is no evidence of a causal link between hypothyroidism and the development of streptococcal sepsis (OR_IVW_: 0.965, 95% CI: 0.825 to 1.128, *P* = 0.651) and puerperal sepsis (OR_IVW_: 0.988, 95% CI: 0.908 to 1.074, *P* = 0.775). Additionally, the research showed no causal association between hyperthyroidism and streptococcal sepsis (OR_IVW_: 1.032, 95% CI: 0.930 to 1.145, *P* = 0.556), puerperal sepsis (OR_IVW_: 0.954 95% CI: 0.815 to 1.115, *P* = 0.552), asthma-associated pneumonia or sepsis (OR_IVW_: 1.031, 95% CI: 0.946 to 1.123, *P* = 0.484), and other sepsis (OR_IVW_: 1.060, 95% CI: 0.987 to 1.139, *P* = 0.108). (**Supplementary Table S2**)

### Reverse MR analyses

The reverse MR results indicated that there was no reverse causality between hypothyroidism and asthma-related pneumonia or sepsis (OR_IVW_: 0.887, 95% CI: 0.754 to 1.044, *P* = 0.149), and other sepsis (OR_IVW_: 0.964, 95% CI: 0.837 to 1.109, *P* = 0.606). (**Supplementary Table S2**)

### Sensitivity analyses

Sensitivity analysis was performed on the results of MR analyses of hypothyroidism and asthma-associated pneumonia or sepsis, and hypothyroidism and other sepsis, to assess the robustness of the results from the MR analyses.

Firstly, the results of MR analyses of hypothyroidism combined with asthma-associated pneumonia or sepsis were explored utilizing the MR Egger intercept versus the MR PRESSO global test, demonstrating no horizontal pleiotropy between the IVs and no outliers were found (*P* > 0.05). The IVW and the MR Egger regression method manifested no heterogeneity between the IVs (*P* > 0.05). Subsequently, sensitivity analysis was conducted on the results of the MR analyses of hypothyroidism and other sepsis, and analyses utilizing the MR Egger intercept and the MR-PRESSO global test demonstrated that there was no horizontal pleiotropy between the IVs and no outliers were found (*P* > 0.05). Analyses utilizing the IVW and the MR Egger revealed that there was no heterogeneity between the IVs (*P* > 0.05).

The leave-one-out analysis was employed to conduct sensitivity analysis to determine whether the observed causal associations relied on a single instrumental variable, demonstrated that the findings from the MR analyses exhibited robustness (**Figures 3C**, **4C**). The funnel plots exhibit a roughly symmetrical distribution, depicting there is no asymmetry in SNP estimation accuracy scales between the IVW and MR Egger (**Figures 3D**, **4D**).

## Discussion

In this research, a two-sample MR method was applied to investigate the causal associations between thyroid dysfunction and streptococcal sepsis, puerperal sepsis, asthma-associated pneumonia or sepsis, and other sepsis, respectively. The findings indicated that hypothyroidism was linked to a higher occurrence of asthma-associated pneumonia or sepsis, and was significantly connected to the incidence of other sepsis. In contrast, hyperthyroidism was not causally linked with the four sepsis subtypes.

Hypothyroidism is characterized by insufficient production of TH, which plays a crucial role in regulating metabolism and immune function. TH promote the development of immune cells from hematopoietic stem cells, and also impact the activity and functionality of immune cells such as macrophages, T cells, and B cells. Consequently, in a state of hypothyroidism, there may be a decrease in the quantity and activity of immune cells, leading to reduced resistance against infections (23, 24) and making individuals more susceptible to bacterial or other pathogenic infections. This heightened susceptibility may elevate the risk of developing sepsis.

TH has a significant impact on cellular oxidative metabolism and plays a crucial role in maintaining vascular balance by promoting positive effects on endothelial and vascular smooth muscle cells. Hypothyroidism, through changes in the morphology and function of target organs and accelerated development of arterial atherosclerosis, compromises blood and oxygen supply to the affected tissues. Consequently, hypothyroidism may have detrimental effects on other physiological functions, such as the cardiovascular and respiratory systems (25, 26). The diminished functionality of these systems can lead to a reduced capacity of the body to respond to infections, thereby increasing the risk of sepsis. Moreover, TH also modulate inflammatory responses. It has been shown (27) that hypothyroidism increases oxidative stress, which exerts its destructive effects through sterile inflammation. It makes the body less able to regulate the inflammatory response, thus predisposing it to an excessive inflammatory response, which may further lead to the development of sepsis.

Huang et al. (28) found that low levels of TH are indicative of weaker constitution in infants, making them more susceptible to developing late-onset bacterial sepsis. Furthermore, hypothyroidism has been recognized as a predictive factor marker for the development of bacterial sepsis in preterm infants. Angelousi et al. (7) discovered that elevated thyroid-stimulating hormone (TSH) levels, lower levels of reverse T3 (rT3), or reduced levels of T3 and T4 have been linked to unfavorable outcomes in individuals suffering from sepsis or septic shock. In a study by Inan et al. (29), the impact of TH supplementation on sepsis criteria and mortality was examined using an experimental sepsis model and revealed that abdominal bacterial loads were decreased in the treated group, and mortality was significantly diminished in the thyroid hormone-treated animals, suggesting that supplementation with TH is beneficial to sepsis benchmarks and reduces mortality in septic rats. Moley et al. (9) indicated that hypothyroidism eliminates the hyperdynamic phase of sepsis and increases susceptibility to sepsis and mortality, whereas T4 replacement after thyroidectomy prevents the increased mortality due to sepsis. Hasselgren et al. (10) also demonstrated that rats with hypothyroidism exhibit impaired response to sepsis. Sepsis led to a reduction in serum T3 levels in both thyroidectomized and sham-thyroidectomized rats, while T3 levels in muscles remained stable or even increased during sepsis. This manifested that augmented uptake of T3 by muscles might contribute to the mechanism underlying the low T3 syndrome observed in sepsis, adding weight to the involvement of TH in altered muscle metabolism during sepsis.

During sepsis, lung injury often occurs as a result of altered metabolism of surfactant, which is necessary for the synthesis of pulmonary surfactant. The normal production of surfactant is dependent on the critical role of T3. Dulchavsky et al. (30) assessed the role of physiologic replacement of T3 during sepsis-induced hypothyroidism on surfactant synthesis and pulmonary structural integrity. Supplementation with T3 during sepsis preserved the histological integrity of lung tissue and was beneficial for ameliorating sepsis-induced pulmonary dysfunction. Another study by Dulchavsky et al. (31) revealed that T3 treatment significantly improved respiratory dynamics in septic animals. In the late stage of sepsis, T3 treatment also enhanced lung elasticity. This indicates that T3 treatment can protect respiratory function in septic rats from both a respiratory dynamics and compliance standpoint. Additionally, supplementing with T3 demonstrated a safeguarding influence on the intestinal barrier in septic rats (32). Our research employed MR analysis, utilizing data from large-scale cohort studies or global genome studies, which have larger sample sizes and better control for confounders, thereby improving the reliability of causal inferences.

Several limitations of this study include the following. Firstly, the results of the MR analyses are mainly based on European populations, and the causality obtained may be ethnically biased, limiting the extrapolation of the results. Furthermore, the SNPs used for analysis may be associated with other traits due to genetic polymorphisms, introducing confounding biases that could affect the interpretation of causality, although we did not find pleiotropy and heterogeneity between the two positive result outcomes by sensitivity analyses. Additionally, since the strength of IVs is influenced by the sample size of the GWAS, conducting a larger-scale genomic analysis is necessary to identify more genetic variations that can be used as IVs in the MR study.

## Conclusion

Taken together, this study identified a causal link between hypothyroidism and the occurrence of asthma-associated pneumonia or sepsis, and other sepsis, but not with the development of streptococcal sepsis and puerperal sepsis. Moreover, our findings did not reveal any causal association between hyperthyroidism and the four sepsis subtypes. Consequently, this study implies that for patients with hypothyroidism who experience infectious illnesses, it is crucial to closely monitor their progression and effectively intervene to prevent the development of sepsis in a timely manner.

## Data availability statement

Publicly available datasets were analyzed in this study. This data can be found here: https://pubmed.ncbi.nlm.nih.gov/34594039/; https://www.finngen.fi/fi/hyodynna_tuloksia.

## Author contributions

JH: Conceptualization, Methodology, Software, Supervision, Validation, Writing – original draft, Writing – review & editing. LZ: Methodology, Supervision, Validation, Writing – original draft. YL: Formal analysis, Funding acquisition, Validation, Writing – review & editing. XC: Data curation, Validation, Writing – review & editing. YC: Funding acquisition, Supervision, Validation, Writing – review & editing. JY: Conceptualization, Funding acquisition, Investigation, Validation, Visualization, Writing – review & editing.
