# Supplementary material for: Causal association between thyroid dysfunction and sepsis: a two-sample mendelian randomization study
Source: Front Endocrinol (Lausanne). 2024 Mar 22;15:1348248. doi: 10.3389/fendo.2024.1348248 (PMC10995304; doi:10.3389/fendo.2024.1348248)
Supplement: Supplementary file 1 [file DataSheet_1.docx]

**Supplementary Figures**


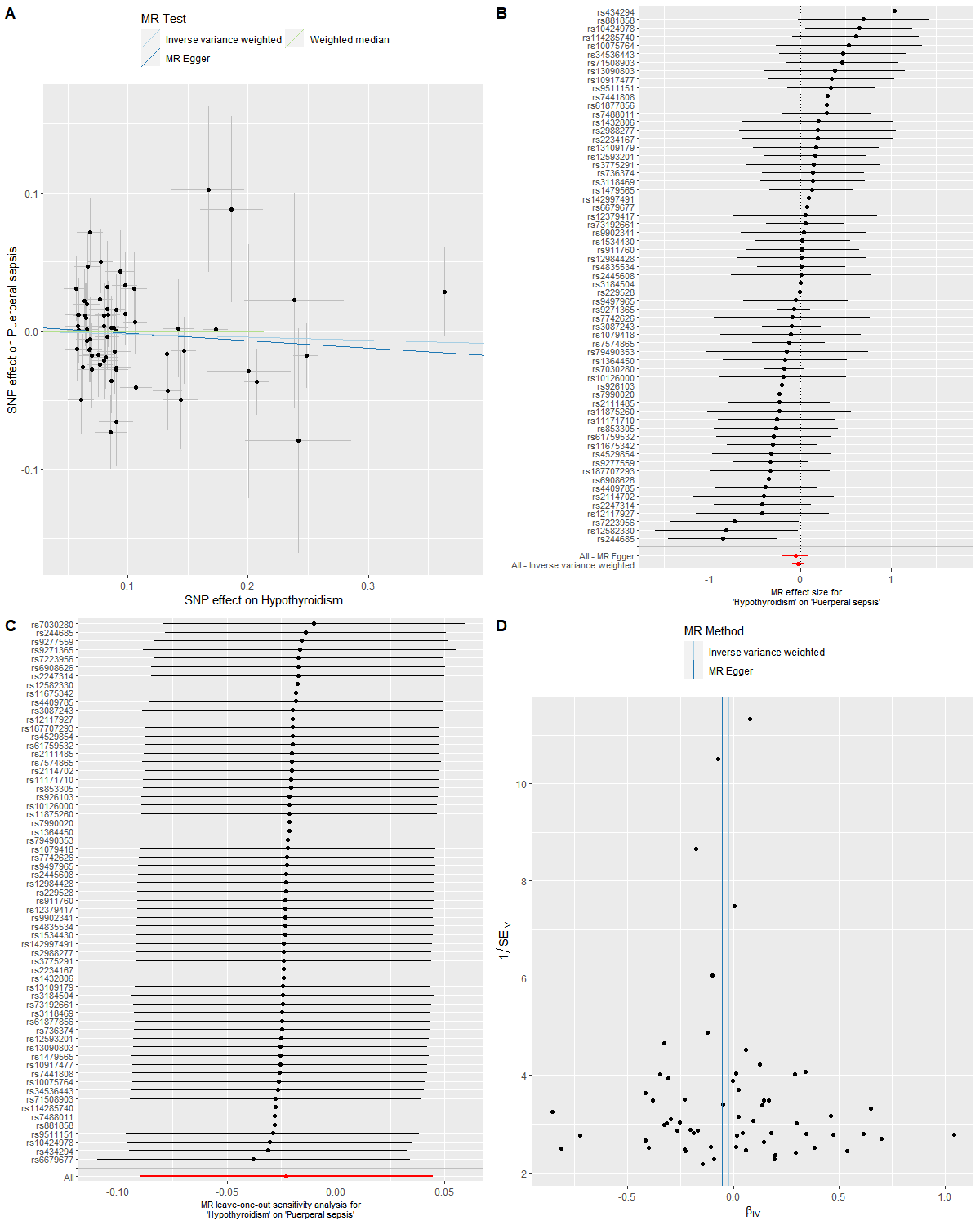
**Supplementary Figure S1** A. Scatter plots of the causality between hypothyroidism and puerperal sepsis by five MR analysis. B. Forest plot of two-sample MR estimates the effects of hypothyroidism on puerperal sepsis. C. The leave-one-out sensitivity plot between hypothyroidism and puerperal sepsis. D. Funnel plots of significant and nominal significant estimates from genetically predicted hypothyroidism on puerperal sepsis.


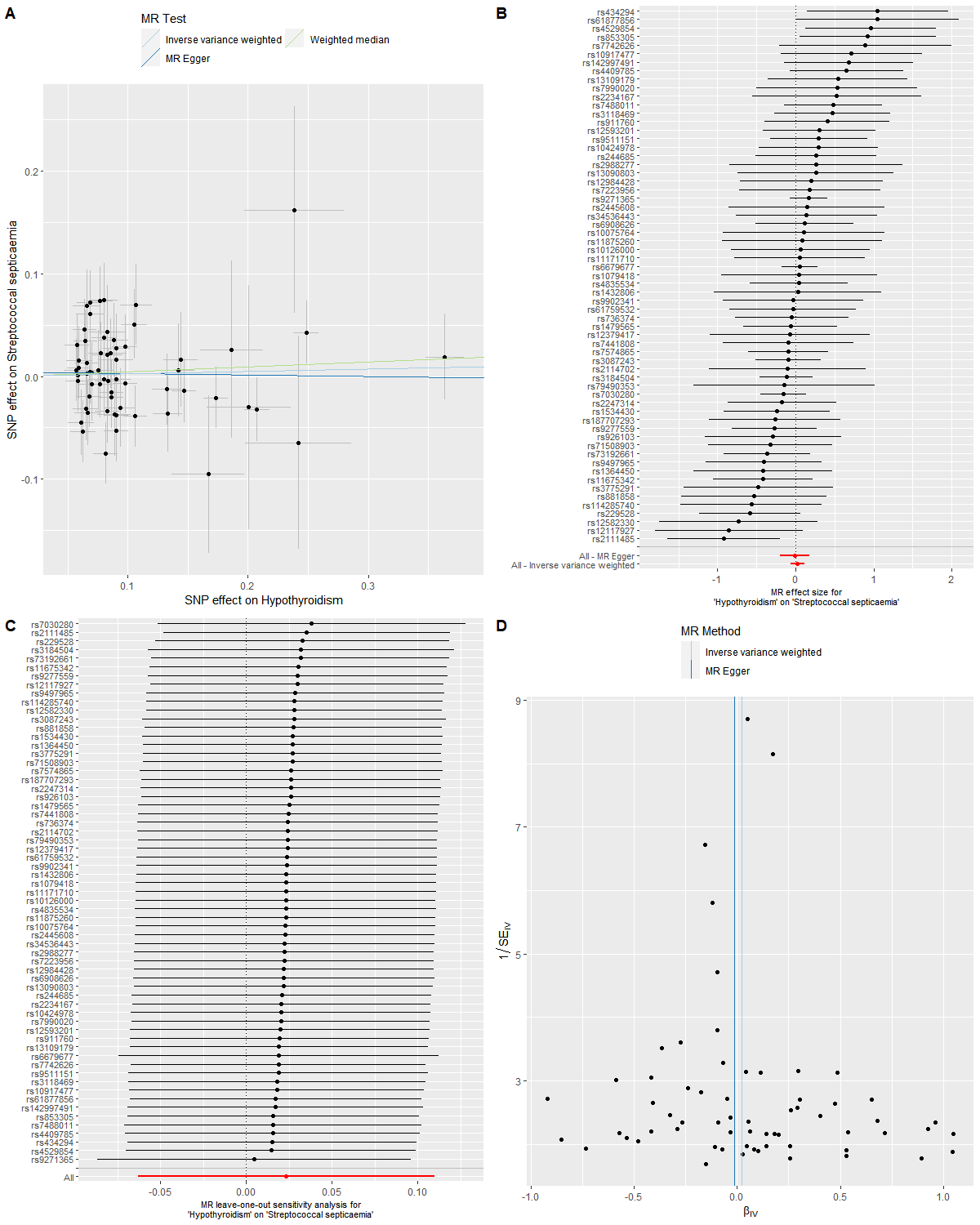
**Supplementary Figure S2** A. Scatter plots of the causality between hypothyroidism and streptococcal sepsis by five MR analysis. B. Forest plot of two-sample MR estimates the effects of hypothyroidism on streptococcal sepsis. C. The leave-one-out sensitivity plot between hypothyroidism and streptococcal sepsis. D. Funnel plots of significant and nominal significant estimates from genetically predicted hypothyroidism on streptococcal sepsis.


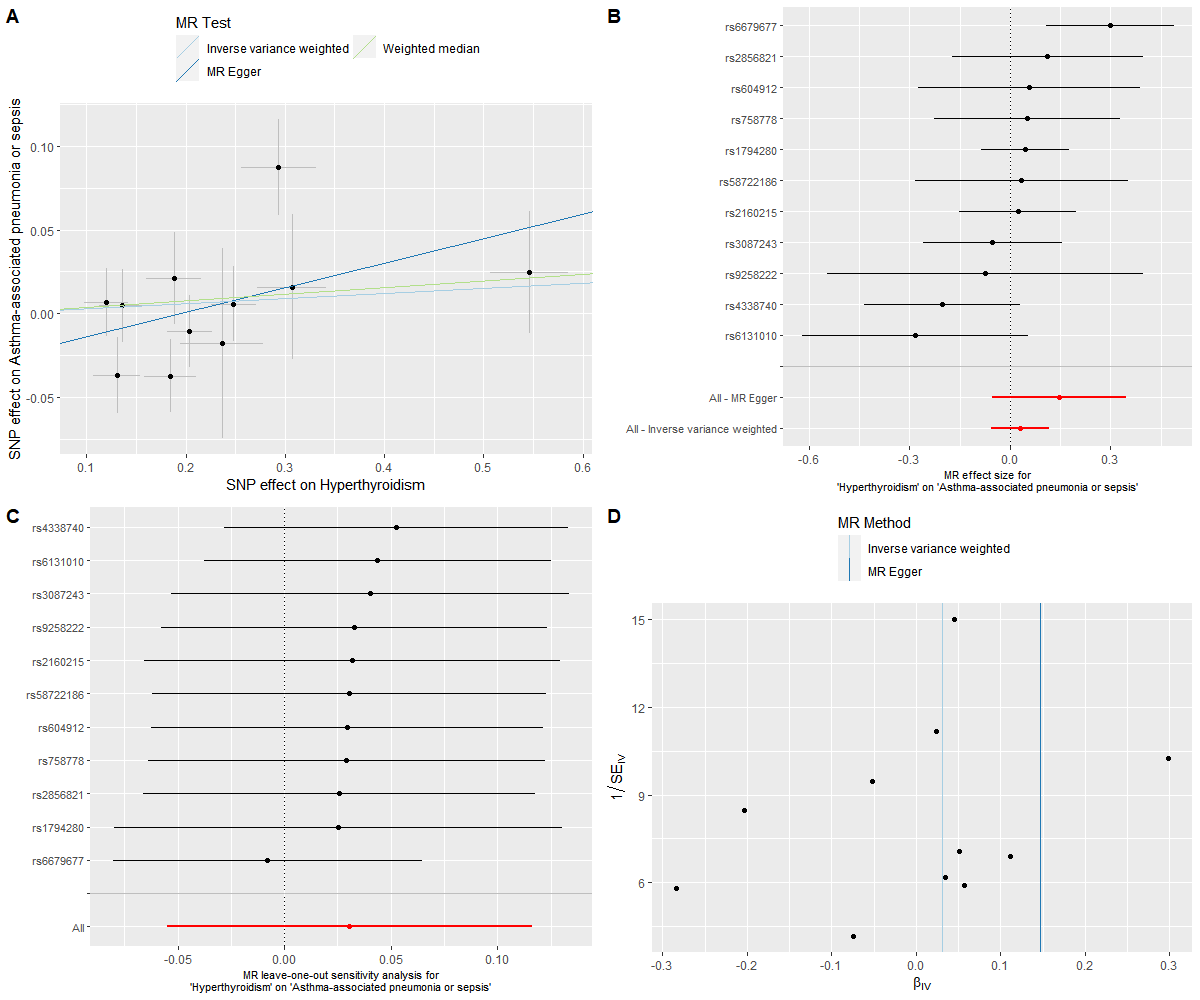


**Supplementary Figure S3** A. Scatter plots of the causality between hyperthyroidism and asthma-associated pneumonia or sepsis by five MR analysis. B. Forest plot of two-sample MR estimates the effects of hyperthyroidism on asthma-associated pneumonia or sepsis. C. The leave-one-out sensitivity plot between hyperthyroidism and asthma-associated pneumonia or sepsis. D. Funnel plots of significant and nominal significant estimates from genetically predicted hyperthyroidism on asthma-associated pneumonia or sepsis.


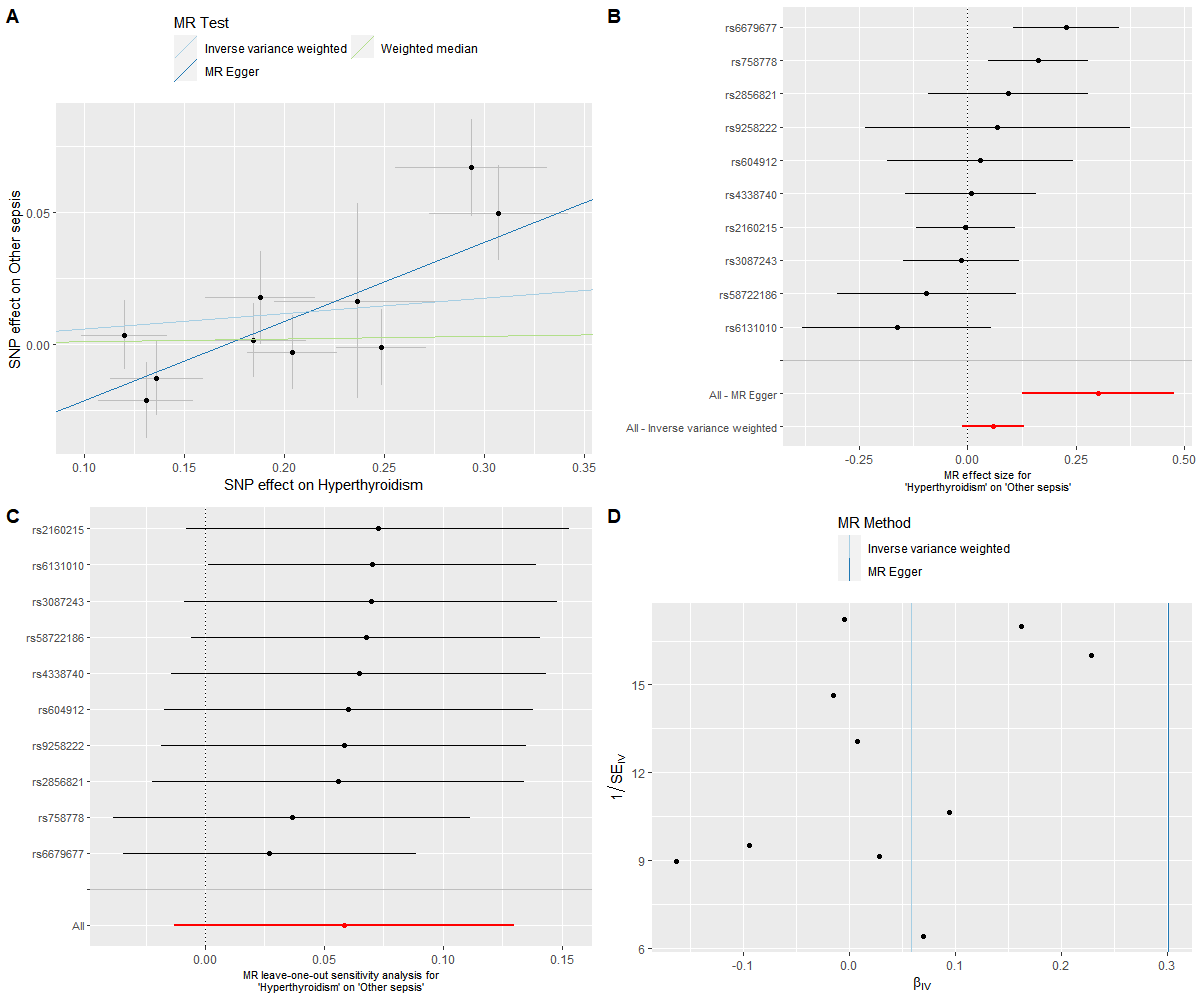


**Supplementary Figure S4** A. Scatter plots of the causality between hyperthyroidism and other sepsis by five MR analysis. B. Forest plot of two-sample MR estimates the effects of hyperthyroidism on other sepsis. C. The leave-one-out sensitivity plot between hyperthyroidism and other sepsis. D. Funnel plots of significant and nominal significant estimates from genetically predicted hyperthyroidism on other sepsis.


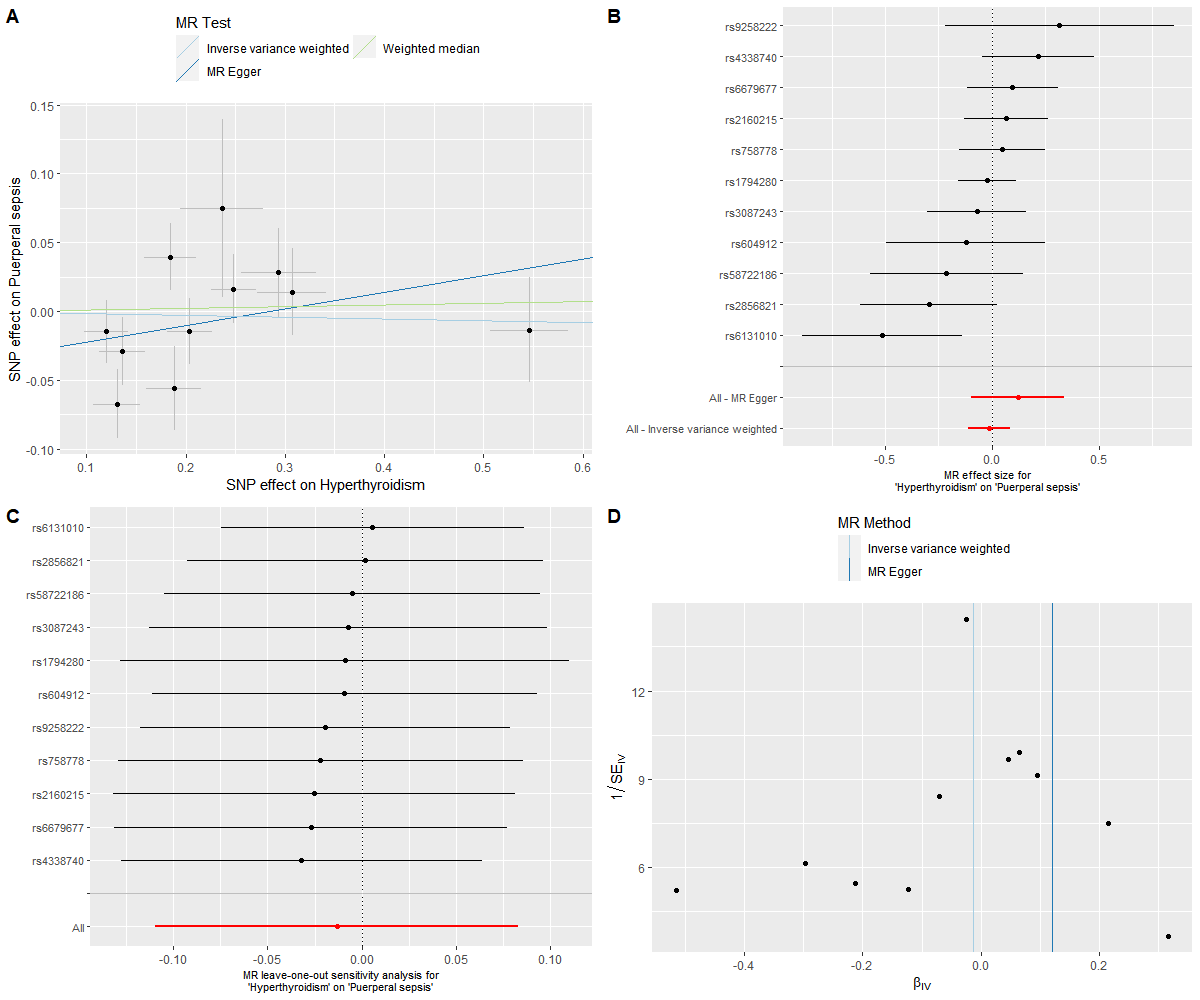
**Supplementary Figure S5** A. Scatter plots of the causality between hyperthyroidism and puerperal sepsis by five MR analysis. B. Forest plot of two-sample MR estimates the effects of hyperthyroidism on puerperal sepsis. C. The leave-one-out sensitivity plot between hyperthyroidism and puerperal sepsis. D. Funnel plots of significant and nominal significant estimates from genetically predicted hyperthyroidism on puerperal sepsis.


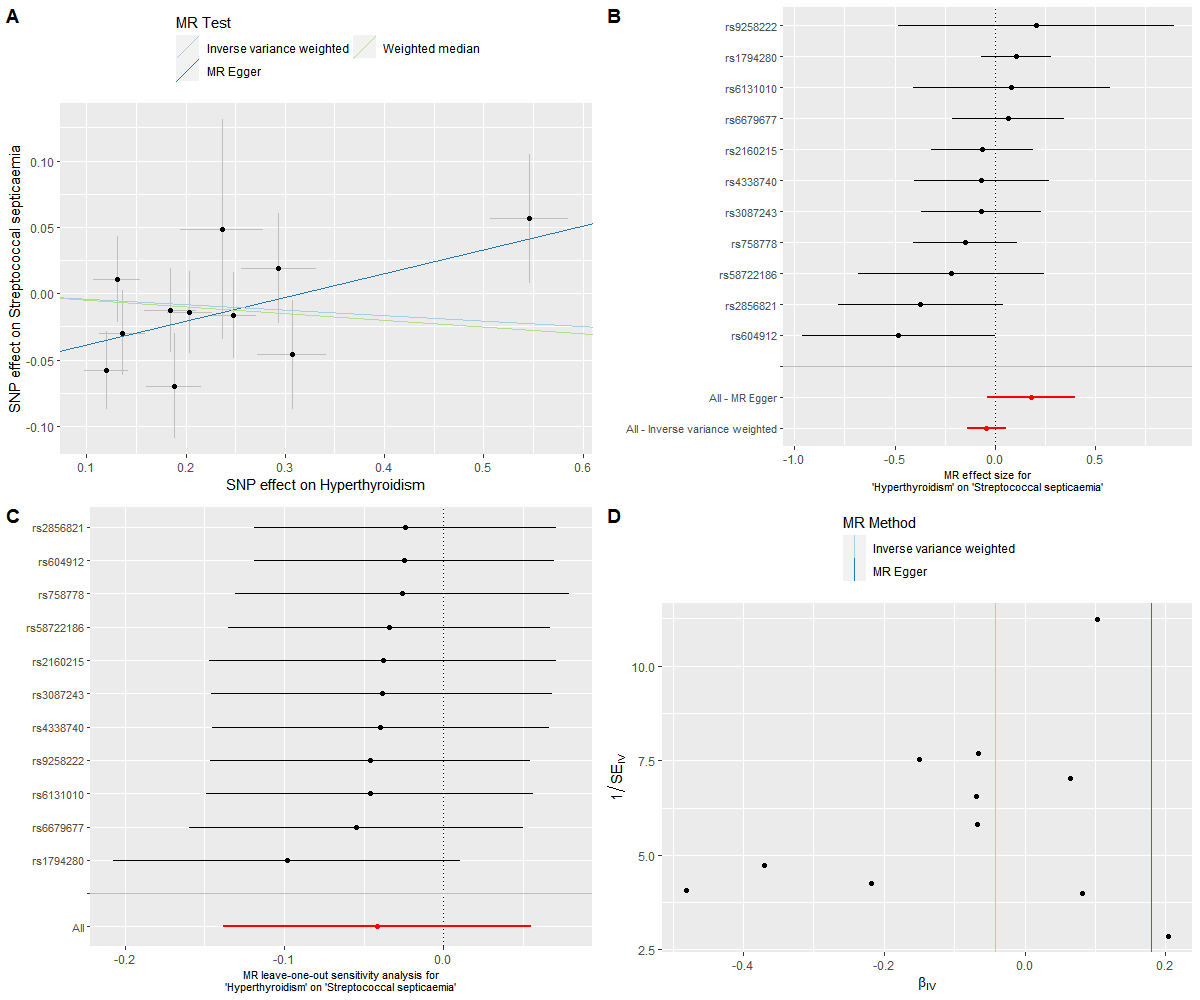
**Supplementary Figure S6** A. Scatter plots of the causality between hyperthyroidism and streptococcal sepsis by five MR analysis. B. Forest plot of two-sample MR estimates the effects of hyperthyroidism on streptococcal sepsis. C. The leave-one-out sensitivity plot between hyperthyroidism and streptococcal sepsis. D. Funnel plots of significant and nominal significant estimates from genetically predicted hyperthyroidism on streptococcal sepsis.
